# Coral skeletons reveal the history of nitrogen cycling in the coastal Great Barrier Reef

**DOI:** 10.1038/s41467-020-15278-w

**Published:** 2020-03-20

**Authors:** Dirk V. Erler, Hanieh Tohidi Farid, Thomas D. Glaze, Natasha L. Carlson-Perret, Janice M. Lough

**Affiliations:** 1 Centre for Coastal Biogeochemistry Research, School of Environment Science and Engineering, Southern Cross University, Lismore, NSW 2480 Australia; 2 Australian Institute of Marine Science, Townsville, QLD 4810 Australia

**Keywords:** Biogeochemistry, Element cycles, Marine chemistry, Ocean sciences, Marine chemistry

## Abstract

Anthropogenic nutrient discharge to coastal marine environments is commonly associated with excessive algal growth and ecosystem degradation. However in the world’s largest coral reef ecosystem, the Great Barrier Reef (GBR), the response to enhanced terrestrial nutrient inputs since European settlement in the 1850’s remains unclear. Here we use a 333 year old composite record (1680–2012) of ^15^N/^14^N in coral skeleton-bound organic matter to understand how nitrogen cycling in the coastal GBR has responded to increased anthropogenic nutrient inputs. Our major robust finding is that the coral record shows a long-term decline in skeletal ^15^N/^14^N towards the present. We argue that this decline is evidence for increased coastal nitrogen fixation rather than a direct reflection of anthropogenic nitrogen inputs. Reducing phosphorus discharge and availability would short-circuit the nitrogen fixation feedback loop and help avoid future acute and chronic eutrophication in the coastal GBR.

## Abstract (web summary)

Coastal pollution degrades ecosystems, but long term impacts are unknown in Australia’s Great Barrier Reef. Using a 333 year record of coral skeleton nitrogen isotopes, Erler and colleagues show that increasing nutrient inputs since European settlement have led to unexpected feedback responses.

## Introduction

**Fig. 1:**
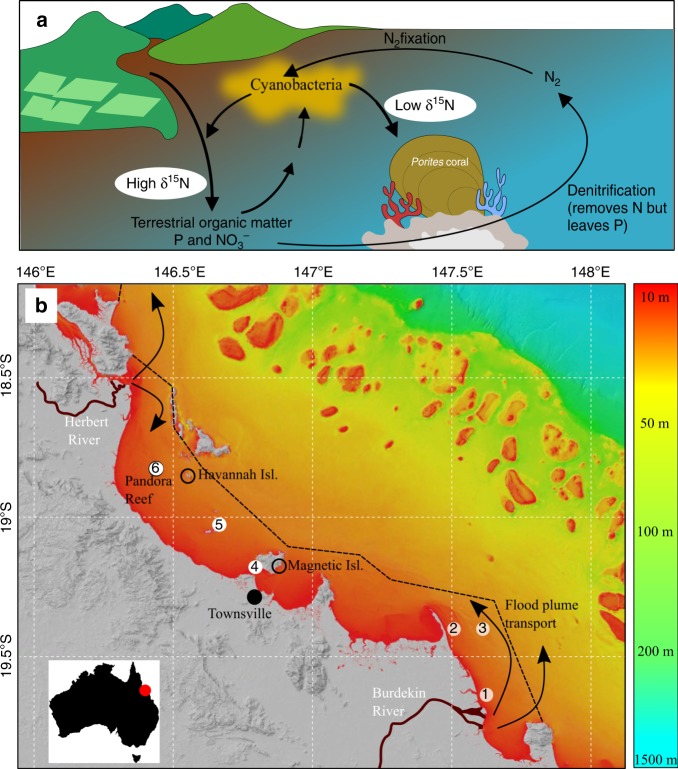
Great Barrier Reef nitrogen cycle and study sites. **a** Simplified conceptual model of nitrogen cycling in the coastal GBR. Terrestrial organic matter, P, and NO_3_^−^ are transported by river runoff. The organic matter and NO_3_^−^ in runoff fuels denitrification but P accumulates in the system. The low N:P ratio stimulates N_2_ fixation which supplies low δ^15^N-N to coastal corals and subsequently decreases their skeletal δ^15^N. The δ^15^N of terrestrial N is higher than that derived from N_2_ fixation. **b** Bathymetric map of the central GBR^67^ showing the location of Havannah Island and Pandora Reef (black circles). The Burdekin and Herbert Rivers, and the generalised direction of flood plume transport (black arrows) are shown. The location of a previous study (Magnetic Island) is also shown. The white numbered dots are the locations of flood plume and coastal water samples collected in March 2019 (sites 1–5), and November 2018 (sites 4–6), respectively. The dotted line encloses the area used to convert literature areal values of N_2_ fixation into N loads per annum.

The global anthropogenic production of nitrogen (N) is now equivalent to the amount of N generated through biological dinitrogen (N_2_) fixation^1^. Furthermore, human activity has more than doubled the pre-industrial input of N into the ocean^2^. While there are many examples of nutrient input to coastal ecosystems leading to algal blooms and eutrophication^3–7^, other systems show a mixed response, including resilience to such inputs^7^. Indeed the discharge of terrestrial nutrients and organic matter can accelerate N loss through microbial processes such as denitrification and anammox^7–9^. In this case phosphorus (P), which is not lost through microbial activity, can accumulate leading to N limitation (i.e. low inorganic N:P ratios)^7,8^. In tropical systems N limitation can promote N_2_ fixation, and this process acts to restore balance to the N:P ratio^7,8,10^ (Fig. 1a). The response of coastal ecosystems to nutrient enrichment therefore is not always a simple cause and effect relationship between nutrient availability and algal growth.

On the Great Barrier Reef (GBR), the world’s largest coral reef ecosystem, numerical estimates^11–14^ and coral skeleton proxy data^15–18^ clearly show that particulate and dissolved nutrient inputs have been increasing since European settlement in the 1850s. This has led to the conclusion that the coastal GBR is not only N replete^19–21^, but probably eutrophic in some parts^20,22–24^. But this is at odds with nutrient budgets for the modern coastal GBR that show strong N deficits (i.e. more N leaving the system than coming in)^25,26^. The discrepancy between N inputs and losses in the coastal GBR, and elsewhere, may simply be due to an over and underestimation, respectively, of rates of denitrification and N_2_ fixation. However in the GBR there is other evidence to suggest that the apparent N deficit is not just a methodological artefact. For example, except during large flood events, concentrations of inorganic N (i.e. NO_3_^−^ and NH_4_^+^) are consistently low (<0.2 μmol L^−1^) in coastal GBR waters^25,27–31^. This may be explained by rapid uptake of inorganic N by phytoplankton, indeed the abundance of phytoplankton is thought to have increased in the coastal GBR as a result of terrestrial nutrient enrichment^24,32^. However, increased phytoplankton abundance has been difficult to confirm against a backdrop of high seasonal variability in chlorophyll concentrations^33,34^ and a general lack of reliable long-term data. In any case, phytoplankton eventually settle and become incorporated into sediment organic matter, which is efficiently processed and subsequently lost via denitrification^26,35,36^.

Denitrification is a major loss pathway for N in the coastal GBR and rates exceed N input through N_2_ fixation (ref. ^26^ and references therein). If enhanced terrestrial discharge is promoting greater denitrification then the preferential loss of N in coastal GBR sediments should manifest as an increase in P availability. This is supported by coastal water column and sediment nutrient concentration data, which consistently shows N:P ratios well below the Refield ratio of 16:1^25,26,28,29,30,31^, and below the average N:P of terrestrial runoff^25^. This has led to the supposition that other than during flood events, the GBR lagoon is N limited for most of the year^30^. Furthermore, nutrient budgets compiled by Furnas, Alongi et al. ^26^ also report that in the wet tropics region of the inshore GBR there is a surplus of P availability but a strong N deficit. This relatively high P availability in the coastal GBR should promote N_2_ fixation^30^. Indeed some of the earliest reports of water quality degradation in the GBR argued that N_2_ fixation has increased as a result of anthropogenic activity^22,37^. However the idea that N_2_ fixation has increased in the coastal GBR as a result of European activity has been contentious and difficult to prove without long-term (i.e. pre-European) records^34^.

So how has N cycling in the coastal GBR responded to increased nutrient input since the 1850s, and how can we address this question without long-term records of N cycle process rates in the coastal GBR? Here, we use the ^15^N/^14^N (i.e. δ^15^N, where δ^15^N = [(^15^N/^14^N)_sample_/(^15^N/^14^N)_air_] − 1) of N trapped in the organic skeletal matrix of massive reef-building corals (hereafter called CS-δ^15^N) to understand how N cycling in the coastal GBR has responded to anthropogenic nutrient discharge since European settlement. The proxy-based approach relies on the fact that the δ^15^N of marine N, which is consumed by corals and recorded in their skeletons^38–40^, is an integrated signal of changes in N sources and availability. Greater N availability as a result of anthropogenic activity should have changed CS-δ^15^N to match the δ^15^N of terrestrial N impacted by fertiliser application and land-clearing. Such modifications can increase the δ^15^N of the residual N pool^41–44^, particularly when surface and groundwater systems are linked^38,45^. Furthermore, N enrichment can cause changes in microbial community composition, and coral physiology, that act to increase δ^15^N (see Table 1 in Erler, Wang et al. ^46^). Therefore, anthropogenic N enrichment should have the net effect of increasing CS-δ^15^N. Alternatively, if N is being lost from the system through greater denitrification, then this should lead to enhanced N_2_ fixation, which would act to reduce CS-δ^15^N as nitrogen fixation reduces the δ^15^N of the water column N pool^47–49^.

For this study, we analysed CS-δ^15^N in four *Porites lutea* coral cores (2–3 samples per year) from two locations, Havannah Island and Pandora Reef, in the central inshore GBR (Fig. 1b). An additional short core from Geoffrey Bay (Magnetic Island) was also analysed to extend a previous coral record from the same reef^46^. As a way of constraining the δ^15^N of N in terrestrial runoff, we also measured the δ^15^N of N in coastal waters before and after a major flood event in 2019. The coral records show a long-term decline in the δ^15^N of the coastal nutrient pool towards the present. When combined with the water sample δ^15^N data, these trends suggest that N_2_ fixation has increased in the coastal GBR since European settlement.

## Results and discussion

### Long-term changes in coral skeleton nitrogen isotopes

**Fig. 2:**
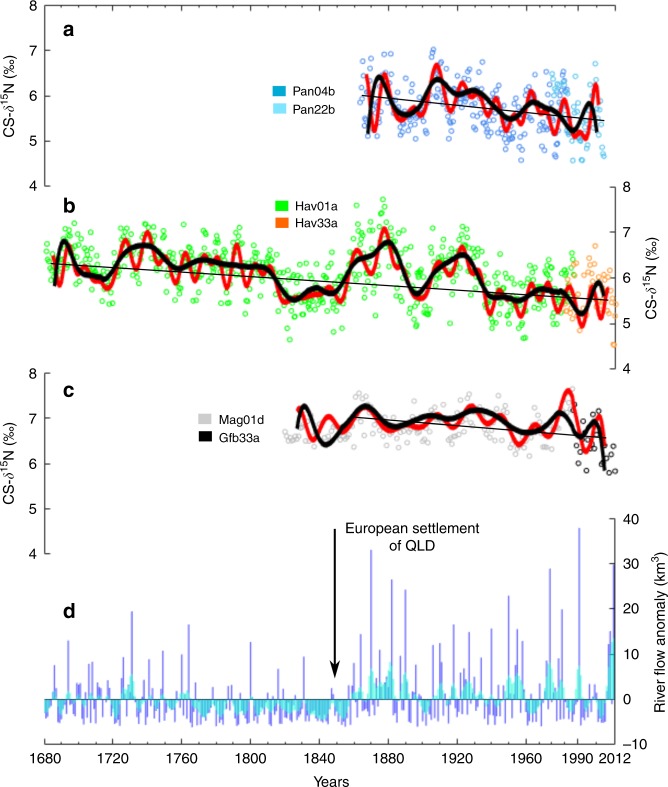
Coral skeleton CS-δ^15^N and reconstructed Burdekin River flow records. **a** Raw data from the two Pandora Reef cores (Pan04b and Pan22b—open circles) overlain with low-pass-filtered CS-δ^15^N (black line = <25 yr frequencies removed) and high-pass-filtered CS-δ^15^N (red line = <10 yr frequencies removed). The straight line is the regression for the composite record (slope = 0.004‰ yr^−1^). **b** Raw data from the two Havannah Reef cores (Hav01a and Hav33a—open circles) overlain with the low and high pass filtered CS-δ^15^N (black and red lines, respectively). The straight line is the regression for the composite record (slope = 0.002‰ yr^−1^). **c** Raw data from the two Magnetic Island cores (Mag01d^46^ and Gfb33a—open circles) overlain with the low and high pass filtered CS-δ^15^N (black and red lines, respectively). The straight line is the regression for the composite record between 1860 and 2011 (slope = 0.002‰ yr^−1^). **d** Reconstructed Burdekin River flow anomalies^50^, data are annual (October–September) water year values (km^3^) normalised to the overall mean flow between 1680 and 2011. Blue lines are the annual values, and blue shading is the 3-year running mean.

The composite CS-δ^15^N records for Havannah Island and Pandora Reef (Fig. 2a, b) were significantly and positively correlated (*r* = 0.56, *p* < 0.001) during the 143-year period over which they overlapped (i.e. 1863–2005) and their long-term average CS-δ^15^N values were also similar (6.0 ± 0.7‰ and 5.8 ± 0.6‰, respectively). Shifts in CS-δ^15^N within each core therefore reflect ecosystem-level changes in external δ^15^N rather than physiological variability within the individual corals. First, we describe the long term changes in the CS-δ^15^N records before examining the reasons that may have caused these changes.

The patterns of CS-δ^15^N variability in corals from both reefs matched reconstructed Burdekin River flow derived from coral luminescence data^50^, indicating that river runoff led to increased CS-δ^15^N, and that dry periods led to decreased CS-δ^15^N (Fig. 2a, b, d). The correlations were generally better when short-term variability was excluded, and prior to 1940 (Supplementary Table 1). These relationships suggest that N enrichment following terrestrial runoff events increases CS-δ^15^N, supporting the notion that bulk terrestrial N has a relatively high δ^15^N, or that increased N availability increases water column N through trophic or metabolic interactions^51^. During dry periods CS-δ^15^N decreases, suggesting the increased contribution of N_2_ fixation to the inshore N pool. The increase in N_2_ fixation during dry periods has been observed in the modern GBR^33^ and fits the hypothesis that in the coastal GBR N can become limiting to the point where diazotrophs can outcompete other phytoplankton^30^.

**Fig. 3:**
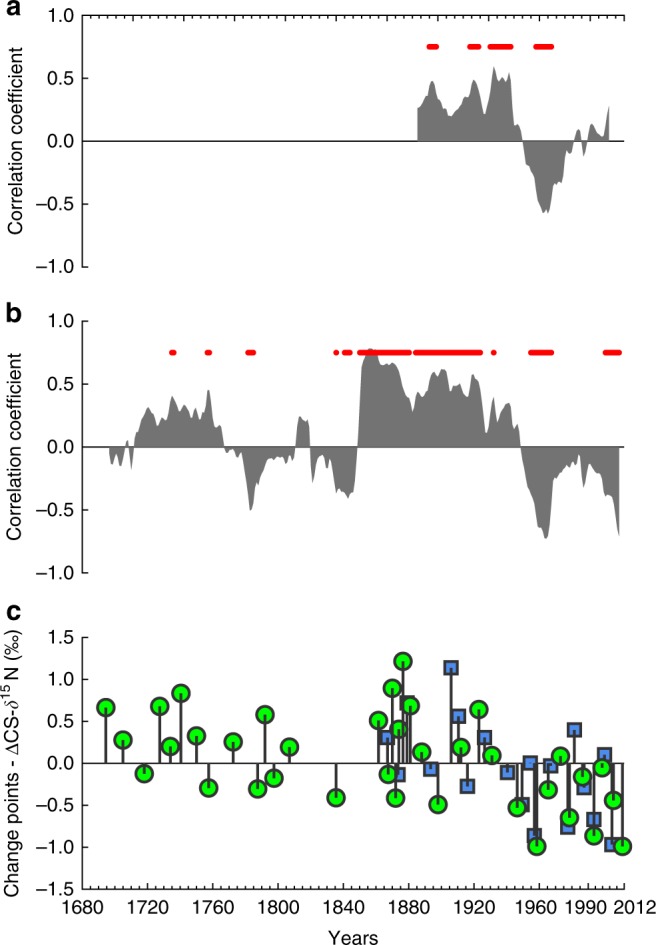
Coral skeleton CS-δ^15^N and Burdekin River flow correlation plots. **a** Correlation coefficients for a moving correlation (30-year window) between the annual Pandora Reef CS-δ^15^N record and Burdekin River flow. Red dots show significance at the 95% level. **b** Correlation coefficients for a moving correlation (30-year window) between the annual Havannah Island CS-δ^15^N record and Burdekin River flow. Red dots show significance at the 95% level. **c** Change points for Pandora Reef (green) and Havannah Island (blue) composite CS-δ^15^N records. Change point values are normalised to the overall mean CS-δ^15^N of each core.

Two lines of evidence suggest that the patterns in N cycling at the study sites have been altered by anthropogenic activity since the 1850s. Firstly, there is a significantly decreasing trend in CS-δ^15^N from 1680 to 2012 (Fig. 2a, b), and secondly there is a shift in the response of CS-δ^15^N to rainfall in the latter half of the 20th century that is not observed during pre-European wet periods (Fig. 3a, b). In the first 100 years of the Havannah Island record (1680–1780) the mean CS-δ^15^N was 6.4 ± 0.5‰, significantly higher (Student’s *t*-test, *p* < 0.001) than the last 100 years of the record (mean CS-δ^15^N of 5.8 ± 0.4‰). The mean CS-δ^15^N between 1940 and 2012 was lower still with a mean of 5.6 ± 0.4‰, representing a 0.8‰ decrease compared to the first 100 years of the record (double the analytical precision of the CS-δ^15^N method). In addition to changes in mean CS-δ^15^N, the Havannah Island record showed a significantly decreasing CS-δ^15^N trend (Mann–Kendall trend test, *p* < 0.001). The Pandora Reef record also had a significant negative trend in CS-δ^15^N between 1863 and 2012 (Mann–Kendall trend test, *p* < 0.001), and while this core does not capture the full extent of climatic variability evident in the Havannah Island record, it can still speak to the changes occurring in the latter half of the 20th century.

Notably, a similar decreasing trend in CS-δ^15^N was not recorded in a previous long core from Magnetic Island (1820–1987), some 60 km south of Havannah Island^46^. While the lack of change in CS-δ^15^N for the Magnetic Island core was used as evidence that there was no N enrichment in the coastal GBR as a result of European settlement^46^, the absence of a decreasing trend warrants further investigation. In the present study we updated the previously published Magnetic Island CS-δ^15^N record with a 25-year short core (1987–2011) from the same reef. A trend test over this new composite record revealed a significant decrease in CS-δ^15^N, but only between 1860 and 2011 (Fig. 2c). In the century before 1860 the central inshore GBR experienced lower than average river runoff (Fig. 2d), which according to the Havannah Island record, resulted in a prolonged decrease in CS-δ^15^N (Fig. 2b). Such a decrease suggests a period of severe N limitation and a greater reliance on N_2_ fixation. Because the Magnetic Island record does not capture the wetter period prior to 1780, we started the trend test from 1860 and not 1820.

The timing of the decrease in CS-δ^15^N in the updated coral record for Magnetic Island is also later than in the Pandora Reef and Havannah Island records. This may in part be due to the removal of the impacts of dredge spoil on Magnetic Island corals, which may have been masking any decrease in CS-δ^15^N. Coral communities on Magnetic Island have been exposed to dredge spoil since the 1880s^52^, but significant efforts to improve the management of dredge spoil discharge only began in the 1990s^53^. Alternatively, the stability of the Magnetic Island record between 1820 and 1987 may be due to the antagonistic effects of increased δ^15^N in terrestrial N runoff (higher CS-δ^15^N) and higher rates of coastal N_2_ fixation (decreased CS-δ^15^N) (see discussion below). Given the proximity of Magnetic Island to the Burdekin River, it may be more influenced by high terrestrial N than the two reefs further north. In any case, taken together, all three coral records from the same coastal region of the GBR suggest a decrease in CS-δ^15^N in the latter half of the 20th century.

In addition to this long-term decreasing trend in CS-δ^15^N, the Havannah Island and Pandora Reef coral records also show a shift in the relationship between CS-δ^15^N and Burdekin River flow between 1860 and 2012. Between 1860 and 1940 CS-δ^15^N and Burdekin River flow anomalies are positively correlated, with peak river flow corresponding with increased CS-δ^15^N and vice versa (Fig. 3a, b). For the Havannah Island core, these correlations are mostly significant. After 1940, both the Havannah Island and Pandora Reef CS-δ^15^N records are poorly, or inversely, correlated with Burdekin River flow anomalies (Fig. 3a, b). A similar pattern of poor and/or inverse correlation between CS-δ^15^N and Burdekin River flow anomalies can also be seen between 1770 and 1850 in the Havannah Island record (Fig. 3b), but this is a particularly dry period with mostly below average river flow (Fig. 2d). Between 1680 and 1770 the average Burdekin River flow anomaly is close to the long-term average (i.e. ~0 km^3^) and there is a positive correlation between CS-δ^15^N and Burdekin River flow anomalies, just like the period between 1860 and 1940. Therefore, the period between 1940 and 2012 stands out from previous periods of comparable Burdekin River runoff. This is confirmed with change point analysis which shows an increase in the number of CS-δ^15^N minima after 1940 in both the Havannah Island and Pandora Reef records, and a reduction in the magnitude of the positive change points after 1940 (Fig. 3c). The sum of our evidence therefore suggests that the CS-δ^15^N is lower after 1940 than during any other previous period of comparable river flow.

### Reasons for decreased CS-δ^15^N in the inshore GBR

The decrease in CS-δ^15^N in the latter half of the 20th century implies that a new source of N with a lower δ^15^N has been added to the coastal GBR N budget. The two possible sources are N_2_ fixation, or terrestrial N that has been modified by the addition of fertiliser (which can have a low δ^15^N^54^). Rainfall is included as a terrestrial source as it deposits atmospheric N back on land which then flows to the coastal GBR. For the Burdekin River catchment the δ^15^N of undisturbed forest soils is ~6.3‰, however modified soils in the modern Burdekin River catchment have an average δ^15^N of ~5.8‰, possibly reflecting the addition of artificial fertilisers since the 1930s^55^. Therefore, the observed decreases in CS-δ^15^N in the coral records could simply be attributed to an increase in the amount of fertiliser used in the Burdekin River catchment, since European settlement (which decreases the δ^15^N of terrestrial N). However, directly linking CS-δ^15^N with terrestrial δ^15^N ignores the complex modifications in isotope abundances that can occur in agricultural systems, particularly when surface and groundwater are linked^38,45^, like they are in the Burdekin River catchment. We propose that the δ^15^N of terrestrial N reaching the coastal GBR is likely to be higher than the δ^15^N of N in soils, and that even though fertiliser N may have a low δ^15^N, by the time this N has moved through the landscape its δ^15^N has been significantly altered by biological processing.

To constrain the δ^15^N of modern-day terrestrial N reaching the central inshore GBR we compared the δ^15^N of the total dissolved N pool (δ^15^N-TDN) before and after a major flooding event (Supplementary Fig. 1) in the central inshore GBR (Sites 1–6 in Fig. 1). Total dissolved N is a mix of terrestrial dissolved organic N (e.g. from soils), dissolved organic N released from particulate N in the water column, and dissolved inorganic N from surface/groundwater sources. The δ^15^N-TDN therefore integrates the δ^15^N of the different N pools in terrestrial discharge or coastal waters, and also captures the isotopic fractionation that occurs during N transformation between the pools (Supplementary Fig. 2). As confirmation of this we measured the δ^15^N of particulate material before the flooding event (Sites 4–6), finding that it was within 0.5‰ of the δ^15^N-TDN at the same sites (Supplementary Table 2). As such, the δ^15^N-TDN should be a good representation of the δ^15^N of N available to coastal corals.

The measured δ^15^N-TDN in the Burdekin River flood plume (March 2019) ranged from 6.2 ± 0.1‰ at the river mouth, to 4.6 ± 0.6‰ at Site 6 (Supplementary Table 2). Prior to the flood event (November 2018) the average δ^15^N-TDN at Sites 4–6 was 4.1 ± 0.7‰ (Supplementary Table 2). The flood plume sampling occurred about a month after the main discharge event, which means there would already have been significant mixing of coastal water with river runoff. To determine the δ^15^N-TDN of the terrestrial endmember, we used an isotope mixing model of measured concentrations and δ^15^N-TDN in water samples collected before and after the flood event (see Supplementary Note 1). With this approach the δ^15^N-TDN of terrestrial N was calculated to be 8.1 ± 1.1‰, this is higher than the δ^15^N of catchment soils^55^ and of fertiliser N^54^. In general CS-δ^15^N is up to 2‰ higher than its N source, so given that the average CS-δ^15^N was 5.6‰ between 1940 and 2012, the δ^15^N-TDN of terrestrial N would need to be between 3.6‰ and 5.6‰ if it was to be the dominant source of N for corals in that period.

Based on our rather limited assessment, it would appear that the calculated δ^15^N of terrestrial N is too high for it to have caused the decreases in CS-δ^15^N observed in the coral records. Other studies also support the idea that the δ^15^N of terrestrial N has increased with increased application of N to adjacent farming land. For example, the δ^15^N in terrestrial material recovered from a Magnetic Island coral skeleton^46^ increased from 2.5‰ to 9.5‰ between 1820 and 1987. Another study measured the δ^15^N of acid insoluble material (which is effectively terrestrial N) in coral skeletons from the southern coastal GBR, finding values of around 8.5‰, and as high as 13.6‰, during flood events^56,57^. These same studies reported δ^15^N values of particulate N in catchment runoff of up to 9.5‰. Unfortunately there are very few studies reporting the δ^15^N of terrestrial N reaching the coastal GBR, but the evidence both from our study and others support the idea that the δ^15^N of terrestrially derived N is too high to be the driver of reduced CS-δ^15^N observed in the coral records from the central inshore GBR.

**Fig. 4:**
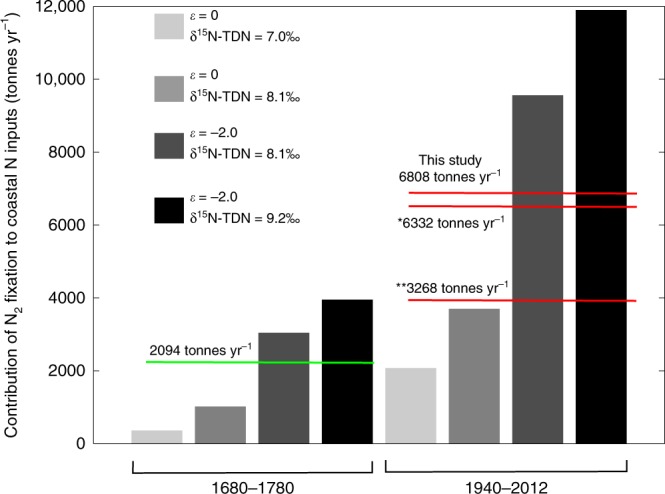
N_2_ fixation (tonnes N yr^−1^) contribution to the inshore GBR. Four scenarios are presented for each time period, these reflect variability in the isotopic fractionation of N during assimilation into coral skeletons (given as *ε*, which is known to vary between 0‰ and −2‰^68^), and the δ^15^N-TDN calculated for terrestrial N (8.1 ± 1.1‰). The four scenarios are the low and high extremes of N_2_ fixation (i.e. *ε* = 0‰/δ^15^N-TDN = 7‰, and *ε* = −2‰/δ^15^N-TDN = 9.2‰, respectively), and N_2_ fixation resulting from *ε* = 0‰/δ^15^N-TDN = 8.1‰, and *ε* = −2‰/δ^15^N-TDN = 8.1‰ (where 8.1‰ is the average δ^15^N-TDN calculated for terrestrial N). The green line is the average of the pre-European period and the red lines are values for the modern GBR. The * value is from Messer, Brown et al. ^31^, and the ** value is the average of Bell, Elmetri et al. ^58^ and Furnas, Alongi et al. ^26^.

The other possible driver of reduced CS-δ^15^N in the Havannah Island, Pandora Reef, and Magnetic Island coral records is N_2_ fixation. To try and quantify the amount of N entering the coastal GBR through N_2_ fixation we developed a second isotope mixing model (see Supplementary Text 2 for a full description). The model assumes that the N available for consumption by coastal corals comes from both terrestrial N inputs (measured as δ^15^N-TDN, see above) and N_2_ fixation. The average CS-δ^15^N value between 1940 and 2012 for the Havannah Island coral was used to determine the contribution of these two sources to the coastal N pool (after accounting for fractionation between the coral and the N source). Outputs from the mixing model show that between 1940 and 2012, the minimum and maximum possible contributions of N_2_ fixation to the inshore N pool are 17% and 55% (Fig. 4). Using the average δ^15^N-TDN value for the flood plume waters of 8.1‰, and accounting for fractionation during uptake of N by corals, N_2_ fixation was constrained to within 27% and 50% of annual N inputs at the study site (Fig. 4).

While our data shows that the proportional contribution of N_2_ fixation to the coastal N pool has increased with anthropogenic inputs, it cannot tell if the rate of N_2_ fixation has increased. For instance, if terrestrial N is being preferentially lost after discharge, then the N from N_2_ fixation may make up a larger fraction of the coastal N pool without the rate of N_2_ fixation actually increasing over time. To convert the fractional contributions into actual amounts of N entering the system through N_2_ fixation, we used the modelled terrestrial N input values of Waters, Carroll et al. ^14^ for the Burdekin and Herbert Rivers (Supplementary Note 3). We estimate that the average annual input of N from N_2_ fixation to the central inshore GBR between 1940 and 2012 ranged between 2073 and 11,897 tonnes yr^−1^, with an average value of 6808 tonnes yr^−1^ (Fig. 4). This is the same order of magnitude as N_2_ fixation estimates from Furnas, Alongi et al. ^26^ (3225 tonnes yr^−1^, which includes water column and sediment rates), the range of values reported by Bell, Elmetri et al. ^58^ (average of 3311 tonnes yr^−1^, water column rates only), and the estimates of Messer, Brown et al. ^31^ (6332 tonnes yr^−1^, water column only). Note that our estimate of N_2_ fixation is calculated as a proportion of the annual terrestrial N load debouched by the Burdekin and Herbert Rivers, whereas the reported literature rates are up-scaled from areal or volumetric rates (see Supplementary Note 3 for a description of the scaling procedure). Furthermore, the literature values relate to coastal regions that may not include our study site. Nevertheless, the similarity between our rates and literature values provides some vindication of our model calculations.

Next we calculated the annual rate of N_2_ fixation in the first 100 years of the record. This period was selected because it is the only interval prior to European settlement that has comparable, albeit lower, rainfall to the modern GBR. To perform the mass balance calculation we used the same mixing model as for the modern GBR, but adjusted the CS-δ^15^N to match the average value between 1680 and 1780 (i.e. 6.4‰). We assumed that the δ^15^N of terrestrial discharge (δ^15^N-TDN) was the same as the modern GBR (i.e. between 7‰ and 9.2‰), which actually overestimates the pre-European rate (because δ^15^N-TDN was probably lower). Similar to the calculation for the modern GBR, we used published estimates of annual terrestrial N inputs prior to European settlement^14^ to deduce a value for N produced through N_2_ fixation (Fig. 4).

The average modelled estimate of N_2_ fixation for the central inshore GBR between 1680 and 1780 was 2094 tonnes yr^−1^ (green line in Fig. 4). This is lower than estimates for the modern GBR (red lines in Fig. 4). Even acknowledging the wide range in the N_2_ fixation estimates, it appears likely that the quantity of N imported into the coastal GBR has increased since pre-European times. This represents the first evidence that N_2_ fixation in the coastal GBR is higher now than prior to European settlement. We hypothesise that increased terrestrial runoff has increased denitrification and decreased the N:P of the coastal GBR since European settlement. This in-turn has manifested as an increase in the amount of N_2_ fixation now occurring in coastal waters.

### Implications for coastal nutrient management in the GBR

Studies on N_2_ fixation conducted in the early 1990s reported increased rates in the coastal GBR relative to the 1930s^22,23^. The increase in N_2_ fixation was thought to import new N and organic matter into the system, potentially stimulating inorganic N availability and enhanced primary production. As such, the observed increases in N_2_ fixation were used as evidence that the GBR was heading towards widespread eutrophication. However, assessments of coastal primary production, paleo-reconstructions, or large-scale N budgets for the GBR do not support this^26,34,59^. Nevertheless, these early N_2_ fixation studies do raise a crucial point, what will happen to the new N imported into the coastal GBR through enhanced N_2_ fixation? From a benthic biogeochemical perspective, an increase in organic N delivery to sediments following N_2_ fixation is likely to fuel sediment anoxia and enhance denitrification, perpetuating N limitation. As P continues to accumulate, eventually the amount of N entering through N_2_ fixation will overwhelm the denitrification capacity of the system, at which point water column DIN availability will increase and classic eutrophic conditions may take hold. Current estimates show that denitrification rates exceed N_2_ fixation rates in the inshore GBR^26^, but it is not clear how long this will persist. While much of this discussion is speculative, it does highlight the importance of reducing terrestrial P discharge to the coastal GBR. Our data suggests that reducing terrestrial P discharge and availability would short-circuit the N_2_ fixation feedback loop and help avoid future acute and chronic eutrophication in the coastal GBR.

## Methods

### Study sites and sample details

*Porites lutea* coral cores from Havannah Island, Pandora Reef, and Magnetic Island (Geoffrey Bay) were selected from the Australian Institute of Marine Sciences Coral Core Archive. The Havannah Island cores cover the period from 1680 to 2012, and the Pandora Reef cores spanned from 1863 to 2005. As such the CS-δ^15^N record is sufficiently long to separate the influence of changing rainfall patterns associated with the end of the Little Ice Age^50^, and the onset of global warming^60^, from the effects of local anthropogenic activity on N dynamics in the central inshore GBR. The chosen reef systems lie between the Burdekin and Herbert Rivers which are ranked 1 and 3, respectively, in terms of average annual freshwater discharge to the GBR^25^. The selected inshore reefs are also considered to be relatively unaffected by oceanic upwelling from the Coral Sea. Therefore, while our study is based on only one small region of the GBR, the coral collection sites are sandwiched between these two large river systems and our findings are broadly applicable to other high input inshore regions of the GBR.

One long core (>100 years old) and one shorter core (<50 years old) from Havannah Island and Pandora Reef were selected for analysis from the AIMS coral core archive (core details are given in Supplementary Table 3). One short core was selected from Geoffrey Bay (Magnetic Island) to extend a previous coral record from the same reef^46^. Coral carbonate powders (~100 mg) were collected from ultrasonically cleaned skeletal slabs using a small drill and a 2 mm diameter carbide engraving bit along the central growth axis. Samples were collected from each high and low density band (i.e. two samples per year of growth). Where annual skeletal extension was >10 mm, additional samples were collected from the low density bands (i.e. minimum of two and maximum of three samples per year of growth). The ages of the skeletal bands were based on previous chronologies of the same cores^61,62^.

### Analysis details

Following collection, skeletal powders were ground with an agate pestle and mortar (to <63 μm) and transferred to a 15 mL centrifuge tube for chemical cleaning with ultrapure NaClO bleach. The detailed method for obtaining intra-crystalline CS-δ^15^N from cleaned coral skeletal material is based on the persulfate oxidation of liberated organic material to NO_3_^−^ and subsequent measurement of its δ^15^N after conversion to N_2_O^51^. Analysis of the δ^15^N-N_2_O produced from the coral powders was performed with a Thermo Delta V Plus Isotope Ratio Mass Spectrometer (IRMS). Nitrous oxide was concentrated with a custom built purge and trap system coupled to the IRMS via a Thermo GasBench II interface. All CS-δ^15^N analyses were performed in triplicate and the analytical precision was 0.4‰. Water samples from the 2018/2019 Burdekin River flood plume, collected by AIMS staff, and water samples from Sites 4 to 6 (November 2018), were collected from 1 and 5 m depth at each site using a Niskin water sampler. All water samples were analysed for total dissolved nitrogen concentration (flow injection analysis), and the δ^15^N of total dissolved nitrogen^63,64^. The δ^15^N and N content of particulate N trapped on pre-combusted glass fibre filter paper was determined on samples (8–24 L) collected from Sites 4 to 6 (November 2018). Analysis was performed on an elemental analyser coupled to an IRMS.

Trend analysis was performed with the Mann–Kendall trend test^65^ after the composite CS-δ^15^N record (i.e. combined raw data from both cores at each site) had been discretised into 0.5 year bins. Comparisons of mean CS-δ^15^N between different time periods was done with Student’s *t*-test. Change point analysis was used to identify abrupt increases or decreases in the composite discretised (0.5 year bins) CS-δ^15^N records. The minimum threshold (i.e. the minimum improvement in total residual error for each change point^66^) was set to 0.4. Change point analysis calculates the period over which a significant change occurs and the mean CS-δ^15^N over that period. Change point values were normalised to the long-term mean in each composite record (i.e. a negative change point represents a decrease relative to the long-term mean).

## Supplementary information

Supplimentary Information

## Data Availability

Coral isotope data is available as an excel file on request to the corresponding author.

## References

[1] Fowler D (2013). The global nitrogen cycle in the twenty-first century. Philos. Trans. R. Soc. B.

[2] Gruber N (2008). The marine nitrogen cycle: overview and challenges. Nitrogen Mar. Environ..

[3] Carpenter SR (1998). Nonpoint pollution of surface waters with phosphorus and nitrogen. Ecol. Appl..

[4] Cloern JE (2001). Our evolving conceptual model of the coastal eutrophication problem. Mar. Ecol.-Prog. Ser..

[5] Diaz RJ, Rosenberg R (2008). Spreading dead zones and consequences for marine ecosystems. Science.

[6] Vitousek PM (1997). Human alteration of the global nitrogen cycle: sources and consequences. Ecol. Appl..

[7] Paerl HW, Piehler MF (2008). Nitrogen and marine eutrophication. Nitrogen Mar. Environ..

[8] Paerl HW (2018). Why does N-limitation persist in the world’s marine waters?. Mar. Chem..

[9] Arrigo KR (2004). Marine microorganisms and global nutrient cycles. Nature.

[10] Paerl HW (2016). It takes two to tango: when and where dual nutrient (N & P) reductions are needed to protect lakes and downstream ecosystems. Environ. Sci. Technol..

[11] Kroon FJ (2012). River loads of suspended solids, nitrogen, phosphorus and herbicides delivered to the Great Barrier Reef lagoon. Mar. Pollut. Bull..

[12] Wooldridge S, Brodie J, Furnas M (2006). Exposure of inner-shelf reefs to nutrient enriched runoff entering the Great Barrier Reef Lagoon: post-European changes and the design of water quality targets. Mar. Pollut. Bull..

[13] McKergow LA, Prosser IP, Hughes AO, Brodie J (2005). Regional scale nutrient modelling: exports to the Great Barrier Reef World Heritage Area. Mar. Pollut. Bull..

[14] Waters, D. et al. *Modelling Reductions**of Pollutant Loads due to Improved Management Practices in the Great Barrier Reef Catchments—Whole of GBR*. Technical report. Vol. 1 (Queensland Department of Natural Resources and Mines, Toowoomba, Queensland, 2013).

[15] McCulloch M (2003). Coral record of increased sediment flux to the inner Great Barrier Reef since European settlement. Nature.

[16] Lewis SE, Shields GA, Kamber BS, Lough JM (2007). A multi-trace element coral record of land-use changes in the Burdekin River catchment, NE Australia. Palaeogeogr. Palaeoclimatol. Palaeoecol..

[17] Alibert C (2003). Source of trace element variability in Great Barrier Reef corals affected by the Burdekin flood plumes. Geochim. Cosmochim. Acta.

[18] Mallela J, Lewis SE, Croke B, Ferse SC (2013). Coral skeletons provide historical evidence of phosphorus runoff on the Great Barrier Reef. PLoS ONE.

[19] Kroon FJ, Thorburn P, Schaffelke B, Whitten S (2016). Towards protecting the Great Barrier Reef from land-based pollution. Glob. Change Biol..

[20] Brodie J, Devlin M, Haynes D, Waterhouse J (2010). Assessment of the eutrophication status of the Great Barrier Reef lagoon (Australia). Biogeochemistry.

[21] Fabricius KE (2005). Effects of terrestrial runoff on the ecology of corals and coral reefs: review and synthesis. Mar. Pollut. Bull..

[22] Bell P (1992). Eutrophication and coral reefs—some examples in the Great Barrier Reef lagoon. Water Res..

[23] Bell PR (1991). Status of eutrophication in the Great Barrier Reef lagoon. Mar. Pollut. Bull..

[24] Bell PR, Lapointe BE, Elmetri I (2007). Reevaluation of ENCORE: support for the eutrophication threshold model for coral reefs. AMBIO: Am. J. Hum. Environ..

[25] Furnas M (2003). *Corals and Catchments-terrestrial Runoff to the Great Barrier Reef*.

[26] Furnas M, Alongi D, McKinnon D, Trott L, Skuza M (2011). Regional-scale nitrogen and phosphorus budgets for the northern (14°S) and central (17°S) Great Barrier Reef sheld ecosystem. Cont. Shelf Res..

[27] Andrews JC, Gentien P (1982). Upwelling as a source of nutrients for the Great Barrier Reef ecosystems: a solution to Darwin’s question?. Mar. Ecol.-Prog. Ser..

[28] Furnas M, Mitchell A (1996). Nutrient inputs into the central Great Barrier Reef (Australia) from subsurface intrusions of Coral Sea waters: a two-dimensional displacement model. Cont. Shelf Res..

[29] Blondeau-Patissier D (2018). Phenology of Trichodesmium spp. blooms in the Great Barrier Reef lagoon, Australia, from the ESA-MERIS 10-year mission. PloS ONE.

[30] Schaffelke B, Carleton J, Skuza M, Zagorskis I, Furnas MJ (2012). Water quality in the inshore Great Barrier Reef lagoon: implications for long-term monitoring and management. Mar. Pollut. Bull..

[31] Messer LF (2017). Diversity and activity of diazotrophs in Great Barrier Reef Surface Waters. Front. Microbiol..

[32] Brodie J, De’Ath G, Devlin M, Furnas M, Wright M (2007). Spatial and temporal patterns of near-surface chlorophyll a in the Great Barrier Reef lagoon. Mar. Freshw. Res..

[33] Revelante N, Gilmartin M (1982). Dynamics of phytoplankton in the Great Barrier Reef Lagoon. J. Plankton Res..

[34] Furnas M, Schaffelke B, David McKinnon A (2014). Selective evidence of eutrophication in the Great Barrier Reef: comment on Bell et al. AMBIO.

[35] Alongi DM, McKinnon AD (2005). The cycling and fate of terrestrially-derived sediments and nutrients in the coastal zone of the Great Barrier Reef shelf. Mar. Pollut. Bull..

[36] Alongi DM, Trott LA, Pfitzner J (2007). Deposition, mineralization, and storage of carbon and nitrogen in sediments of the far northern and northern Great Barrier Reef shelf. Cont. Shelf Res..

[37] Bell PRF, Elmetri I (1995). Ecological indicators of large-scale eutrophication in the Great Barrier Reef lagoon. Ambio.

[38] Erler, D. V., Shepherd, B. O., Linsley, B. K., Lough, J. & Cantin, N. E. Coral skeletons record increasing agriculture related groundwater nitrogen inputs to a South Pacific reef over the past century. *Geophys. Res. Lett.* **45**, 8370–8378 (2018).

[39] Ren H (2017). 21st-century rise in anthropogenic nitrogen deposition on a remote coral reef. Science.

[40] Wang XT (2018). Natural forcing of the North Atlantic nitrogen cycle in the Anthropocene. Proc. Natl Acad. Sci. USA.

[41] Robinson D (2001). δ15N as an integrator of the nitrogen cycle. Trends Ecol. Evol..

[42] Xue D (2009). Present limitations and future prospects of stable isotope methods for nitrate source identification in surface and groundwater. Water Res..

[43] Wells NS, Baisden WT, Horton T, Clough TJ (2016). Spatial and temporal variations in nitrogen export from a New Zealand pastoral catchment revealed by stream water nitrate isotopic composition. Water Resour. Res..

[44] Snider DM, Wagner-Riddle C, Spoelstra J (2017). Stable isotopes reveal rapid cycling of soil nitrogen after manure application. J. Environ. Qual..

[45] Bohlke, J. K., Smith, R. L. & Miller, D. N. Ammonium transport and reaction in contaminated groundwater: application of isotope tracers and isotope fractionation studies. *Water Resour. Res.* **42** (2006).

[46] Erler DV (2016). Nitrogen isotopic composition of organic matter from a 168 year-old coral skeleton: Implications for coastal nutrient cycling in the Great Barrier Reef Lagoon. Earth Planet. Sci. Lett..

[47] Montoya JP, Carpenter EJ, Capone DG (2002). Nitrogen fixation and nitrogen isotope abundances in zooplankton of the oligotrophic North Atlantic. Limnol. Oceanogr..

[48] McClelland JW, Holl CM, Montoya JP (2003). Relating low delta N-15 values of zooplankton to N-2-fixation in the tropical North Atlantic: insights provided by stable isotope ratios of amino acids. Deep-sea Res. Part I.

[49] Wada E, Hattori A (1976). Natural abundance of 15N in particulate organic matter in the North Pacific Ocean. Geochim. Cosmochim. Acta.

[50] Lough J, Lewis S, Cantin N (2015). Freshwater impacts in the central Great Barrier Reef: 1648–2011. Coral Reefs.

[51] Wang XT (2015). Isotopic composition of skeleton-bound organic nitrogen in reef-building symbiotic corals: a new method and proxy evaluation at Bermuda. Geochim. Cosmochim. Acta.

[52] Pringle, A. W. *The History of Dredging in Cleveland Bay, Queensland and its effect on Sediment Movement and on the Growth of Mangroves, Corals and Seagrass* (Great Barrier Reef Marine Park Authority, 1989).

[53] Benson, L., Goldsworthy, P., Butler, I. & Oliver, J. J. *Townsville Port Authority Capital Dredging Works 1993: Environmental Monitoring Program* (Townsville, Qld.: Townsville Port Authority, 1994).

[54] Kendall, C., Elliot, E. M. & Wankel, S. D. *Stable Isotopes in Ecology and Environmental Science* (Blackwell Publishing, 2007).

[55] Bahadori M (2019). A novel approach of combining isotopic and geochemical signatures to differentiate the sources of sediments and particulate nutrients from different land uses. Sci. Total Environ..

[56] Jupiter S (2008). Linkages between coral assemblages and coral proxies of terrestrial exposure along a cross-shelf gradient on the southern Great Barrier Reef. Coral Reefs.

[57] Marion, G. *The Nitrogen Isotopic Composition Of The Organic Matrices Of Coral Skeleton: A Proxy For Historical Nitrogen Provenance In Tropical Coastal Oceans* (University of Queensland, 2007).

[58] Bell P, Elmetri I, Uwins P (1999). Nitrogen fixation by *Trichodesmium* spp. in the Central and Northern Great Barrier Reef Lagoon: relative importance of the fixed-nitrogen load. Mar. Ecol. Prog. Ser..

[59] Johnson J, Perry C, Smithers S, Morgan KM, Woodroffe S (2019). Reef shallowing is a critical control on benthic foraminiferal assemblage composition on nearshore turbid coral reefs. Palaeogeogr. Palaeoclimatol. Palaeoecol..

[60] Abram NJ (2016). Early onset of industrial-era warming across the oceans and continents. Nature.

[61] Lough, J. M. Great Barrier Reef coral luminescence reveals rainfall variability over northeastern Australia since the 17th century. *Paleoceanography* **26** (2011).

[62] Lough JM (2011). Measured coral luminescence as a freshwater proxy: comparison with visual indices and a potential age artefact. Coral Reefs.

[63] Erler DV, Wang XT, Sigman DM, Scheffers S, Shepherd BO (2015). Controls on the nitrogen isotopic composition of shallow water corals across a tropical reef flat transect. Coral Reefs.

[64] Knapp AN, Sigman DM, Lipschultz F (2005). N isotopic composition of dissolved organic nitrogen and nitrate at the Bermuda Atlantic Time‐series Study site. Biogeochemical Cycles.

[65] Hammer Ø, Harper D, Ryan P (2001). Paleontological statistics software: package for education and data analysis. Palaeontol. Electron..

[66] Killick R, Fearnhead P, Eckley IA (2012). Optimal detection of changepoints with a linear computational cost. J. Am. Stat. Assoc..

[67] Beaman, R. *Project 3D-GBR: A high-resolution depth model for the Great Barrier Reef and Coral Sea*. Marine and Tropical Sciences Research Facility (MTSRF) Project 2.5i.1a Final Report, MTSRF. pp. 13 plus Appendix 1 (James Cook University, Cairns, Australia, 2010).

[68] Wang XT (2016). Influence of open ocean nitrogen supply on the skeletal δ 15 N of modern shallow-water scleractinian corals. Earth Planet. Sci. Lett..

