# Supplementary material for: Coral skeletons reveal the history of nitrogen cycling in the coastal Great Barrier Reef
Source: Nat Commun. 2020 Mar 20;11:1500. doi: 10.1038/s41467-020-15278-w (PMC7083840; doi:10.1038/s41467-020-15278-w)
Supplement: Supplementary file 1 — Supplimentary Information [file 41467_2020_15278_MOESM1_ESM.pdf]

Supplementary Information for:

Coral skeletons reveal the history of nitrogen cycling in the coastal Great Barrier Reef

Erler et al.

Supplementary Notes

Supplementary Note 1 - Isotope mixing model – water column

The $\delta^{15}N$ of total dissolved N in coastal water after the flooding event of early 2019 ($\delta^{15}N$-*TOT*) is a mix of the $\delta^{15}N$-TDN from terrestrial runoff ($\delta^{15}N$-*TR*) and the $\delta^{15}N$-TDN in the coastal water prior to flooding ($\delta^{15}N$-*CW*). The following equation can be used to isolate $\delta^{15}N$-*TR*:

$$\delta^{15}N - TOT \times [TOT] = (\delta^{15}N - TR) \times [TR] + (\delta^{15}N - CW) \times [CW] \qquad \text{Equation 1}$$

$$[TOT] = [TR] + [CW] \qquad \text{Equation 2}$$

Where:

$[TOT]$ is the total measured TDN concentration in the flood plume samples

$[TR]$ is the concentration of terrestrial N in the river runoff

$[CW]$ is the concentration of TDN in the coastal water prior to flooding.

The values of $[CW]$ and $\delta^{15}N$-*CW* were measured in November 2018 at 3 inshore sites (Supplementary Table 2) and averaged. The value for [TR] was then calculated by difference. Equation 1 was then solved for $\delta^{15}N$-*TR* for each flood plume sample. The average $\delta^{15}N$-*TR* (with propagated errors) was 8.1 ± 1.1‰.

Supplementary Note 2 - Isotope mixing model - coral

The isotope mixing model calculates the $\delta^{15}N$ of N available in the water column ($\delta^{15}N$-*AvailN*). Corals' will assimilate this N and the CS-$\delta^{15}N$ record is directly related to $\delta^{15}N$-*AvailN* through the variable ε, the isotopic fractionation between the available N and skeletal N (Equation 2). This fractionation value varies between 0% and -2‰ (Erler et al. 2015; Wang et al. 2016; Ren et al. 2017). The model assumes that there are two major sources of N to the inshore GBR reefs, terrestrial runoff and N_2 fixation (Equations 3 and 4). By fixing the $\delta^{15}N$ of terrestrial runoff, and matching the calculated $\delta^{15}N$-*AvailN* against measured CS-$\delta^{15}N$, we were able to constrain the contribution of terrestrial N and N_2 fixation to the N available to the corals during different time periods (Equation 5). The input of N from precipitation and upwelling are considered to be relatively minor for inshore corals (Furnas et al. 2011).

$$(\delta^{15}N - AvailN) = (CS - \delta^{15}N) + \varepsilon \qquad \text{Equation 2}$$

$$(\delta^{15}N - AvailN) = (\delta^{15}N - TR) \times fTR + (\delta^{15}N - N_2) \times fN_2 \qquad \text{Equation 3}$$

$$1 = fTR + fN_2 \qquad \text{Equation 4}$$

$$fTR = \frac{(\delta^{15}N - AvailN) - (\delta^{15}N - N_2)}{(\delta^{15}N - TR) - (\delta^{15}N - N_2)} \qquad \text{Equation 5}$$

Where:

$\delta^{15}N\text{-}AvailN$ = δ^{15}N (‰) of the water column N pool available to the corals

$CS\text{-}\delta^{15}$N = skeletal δ^{15}N (‰)

ε = isotopic fractionation between corals' N source and skeletal N (‰)

$\delta^{15}N\text{-}TR$ = δ^{15}N of N from terrestrial runoff (‰)

fTR = fraction of new N available to inshore corals that is derived from terrestrial runoff

$\delta^{15}N\text{-}N_2$ = δ^{15}N of N from N_2 fixation (‰)

fN_2 = fraction of new N available to inshore corals that is derived from N_2 fixation

For the modern coastal GBR, we used the calculated value of δ^{15}N-TR from Equation 1 (Supplementary Note 1) to represent the δ^{15}N of terrestrial runoff (i.e. 8.1 ± 1.1‰). The δ^{15}N of N produced through N_2 fixation was assigned a value of -1‰. Using the average CS-δ^{15}N from the Havannah Island core between 1940 and 2012 of 5.6‰, and with ε values ranging between 0‰ and -2‰, the minimum and maximum possible contributions of N_2 fixation to the available N pool were 17% (δ^{15}N-TR of 7‰) and 55% (δ^{15}N-TR of 9.2‰) for ε values ranging between 0‰ and -2‰ respectively. Using the average δ^{15}N-TDN of 8.1‰, the contribution of N_2 to new N inputs to the coastal GBR ranged between 27% and 50% (for ε values ranging between 0‰ and -2‰ respectively).

For the first 100 years of the CS-δ^{15}N record (i.e. pre-European settlement) we repeated the calculations using the average CS-δ^{15}N between 1680 and 1780 of 6.4‰. The same δ^{15}N-TR from the modern GBR were used for the pre-European calculation. Given that δ^{15}N-TR has likely risen since European settlement, the contribution of N_2 fixation to the

coastal ocean N pool in the first 100 years of the record is an overestimation. The $\delta^{15}N$ of N produced through N_2 fixation was assigned a value of -1‰. With the endmember $\delta^{15}N$ values set, and using high and low estimates of *$\delta^{15}N$-AvailN* (i.e. ε varying between 0‰ and -2‰), the contribution of terrestrial N and N_2 fixation to the N being assimilated by the corals could be calculated (Figure 4 in main text).

Supplementary Note 3 – Estimating N inputs from N_2 fixation

The model calculations presented above estimate the fractional contribution of N from N_2 fixation to new N entering the coastal GBR, which is then recorded as CS-$\delta^{15}N$. To convert this to an actual amount of N from N_2 fixation added to the water column each year we need to know the total amount of N that is available to the corals before and after European settlement. We cannot estimate this from the coral CS-$\delta^{15}N$ as this only provides a value of the water column $\delta^{15}N$ pool, not its concentration. Instead we must use modelled values of N inputs to the coastal GBR. Using values of combined terrestrial N inputs to the coastal GBR from the Burdekin and Herbert Rivers before and after European settlement (i.e. 4444 tonnes yr^{-1} and 9773 tonnes yr^{-1} respectively (Waters et al. 2013)) we calculated the amount of N inputs from N_2 fixation according to Equation 6.

$$N - N_2 = TotalN \times \frac{fN_2}{(1-fN_2)} \qquad \text{Equation 6}$$

Where:

N-N_2 = annual N from N_2 delivered to the coastal GBR relative to the N discharge from the Burdekin and Herber Rivers (tonnes yr^{-1})

TotalN = the annual N discharge from the Burdekin and Herber Rivers (tonnes yr^{-1})

The annul inputs of N from N_2 fixation presented here are calculated as a fraction of the terrestrial N exports. Previous measurements of N_2 fixation in the coastal GBR are given as areal rates (tonnes km^2, or mmol m^{-2} d^{-1}) (Bell et al. 1999; Furnas et al. 2011), or volumetric rates (nmol L^{-1} d^{-1}) (Messer et al. 2017). To convert these to an annual N input value we estimated the surface area of the inshore GBR (~ 20 km from land) between the Burdekin and Herbert Rivers (3872 km^2) and multiplied the areal rates by this area. For the volumetric conversion we assumed an average depth of 10 m.

Comparing our annual rates with the scaled up literature values is problematic because it assumes that the river discharge affects the same area as is used to scale up the areal rates. However river discharge may extend further than the area enclosed by the dashed line in Fig. 1 (main text). In this case the areal rates would need to be multiplied by a higher km^2 area value.

Supplementary Figures

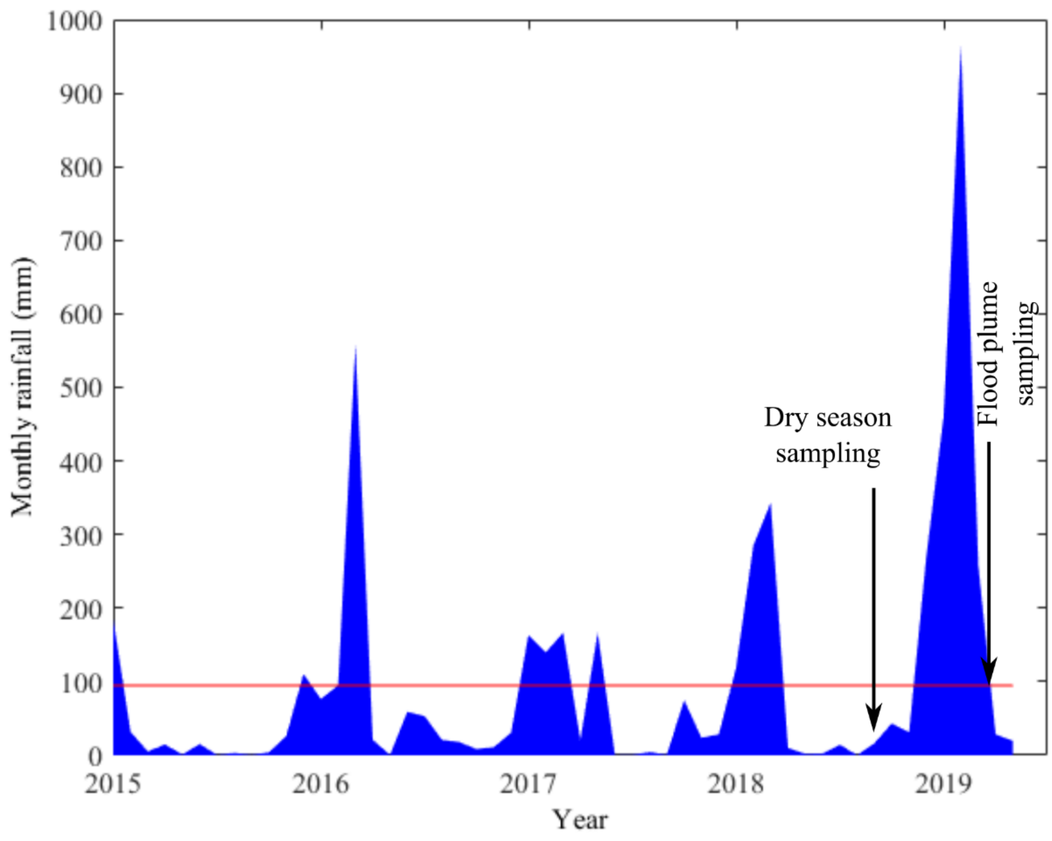

Supplementary Figure 1. Monthly rainfall (mm) in the Townsville region between January 2015 and April 2019. Red line is the long term average for the region (Australian Bureau of Meteorology - http://www.bom.gov.au/jsp/ncc/cdio/wData/wdata?p_nccObsCode=139&p_display_type=dataFile&p_stn_num=032040). The coastal dry season water sampling and the flood plume sampling times are shown.

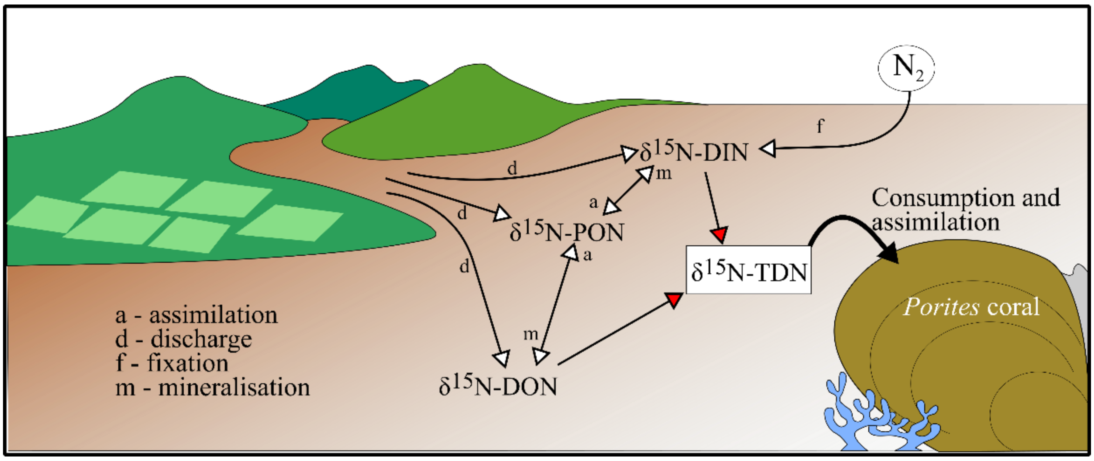

Supplementary Figure 2. Conceptual model showing the nitrogen (N) pools in the coastal GBR and the processes that influence the δ^{15}N of these pools. DIN is dissolved inorganic N, DON is dissolved organic N, PON is particulate organic N, N_2 represents N_2 fixation, and TDN is total dissolved N. The δ^{15}N of TDN integrates the N transformations between the different pools and is assumed to be the δ^{15}N of N that is available to corals.

Supplementary Tables

Supplementary Table 1. Results of Pearson correlation tests between CS-δ^{15}N from Havannah Island and Pandora Reef, and the reconstructed Burdekin River flow of Lough et al. (2015). The coral records are a composite of the different coral cores for that reef (n = 2). Annual data has not been filtered. Other data have had different frequency bands removed, <10 means that data was filtered to remove periods of less than 10 years, <25 means that data was filtered to remove periods of less than 24 years, and <50 means that data was filtered to remove periods of less than 50 years.

Data filtering	Havannah Island CS-δ^{15}N vs Burdekin River flow		Pandora Reef CS-δ^{15}N vs Burdekin River flow	
	r value	Probability	r value	Probability
Annual unfiltered	-	-	0.15	0.06
<10 yr periods	-	-	0.19	0.02
<25 yr periods	-	-	0.44	<0.001
<50 yr periods	0.24	< 0.001	NA	NA

Data filtering	Havannah Island CS-δ^{15}N vs Burdekin River flow prior to 1940		Pandora Reef CS-δ^{15}N vs Burdekin River flow prior to 1940	
	r value	Probability	r value	Probability
Annual unfiltered	0.2	<0.005	0.21	0.06
<10 yr periods	0.43	< 0.001	0.185	.02
<25 yr periods	0.51	< 0.001	0.33	<0.003
<50 yr periods	0.56	< 0.001	NA	NA

Supplementary Table 2. Concentrations and $\delta^{15}N$ of total dissolved N (TDN) and particulate N (PN) in coastal waters in November 2018a (Sites 4, 5, 6 in Figure 1 main text) and concentrations and $\delta^{15}N$ of total dissolved N in a Burdekin River flood plume in March 2019 in the central GBR (sites 1, 2, 3, 5 and 6 in Figure 1 main text). Site ID refers to the locations in Fig. 1 of the main text. The values are the average ± SD for 4 samples (post-flood), and 3 samples (pre-flood).

Pre-flood coastal sampling – November 2018							
Site	Collection date	Collection location (°S, °E)		[TDN] μmol L^{-1}	$\delta^{15}N$ – TDN (‰)	[PN] μmol L^{-1}	$\delta^{15}N$ – PN (‰)
4	12/11/2018	-18.998	146.708	8.8 ± 0.3	4.7 ± 0.6	0.43 ± 0.2	4.2 ± 0.4
5	12/11/2018	-18.817	146.438	10.1 ± 0.6	3.9 ± 0.2	0.60 ± 0.2	3.6 ± 0.2
6	12/11/2018	-19.143	146.785	9.7 ± 0.3	3.2 ± 0.3	0.70 ± 0.3	3.7 ± 0.3

Post-flood coastal sampling – March 2018					
Site	Collection date	Collection location (°S, °E)		[TDN] μmol L^{-1}	$\delta^{15}N$ – TDN (‰)
1	7/03/2019	-19.588	147.582	17.9 ± 0.6	6.2 ± 0.1
2	7/03/2019	-19.366	147.487	16.6 ± 0.4	6.1 ± 0.1
3	13/03/2019	-19.305	147.622	15.0 ± 0.2	5.5 ± 0.3
5	8/03/2019	-18.998	146.708	10.5 ± 0.3	4.7 ± 0.1
6	8/03/2019	-18.817	146.438	10.6 ± 0.2	4.6 ± 0.1

Supplementary Table 3. Coral cores used for CS-$\delta^{15}N$ analysis

Coral core ID	Collection location	Collection year	Years of growth analysed
Hav01a	Havannah Island<br>18.837°S, 146.548°E	1987	1681 – 1987
Hav33b	Havannah Island<br>18.837°S, 146.548°E	2012	1981 - 2012
Pan04b	Pandora Reef<br>18.815°S, 146.436°E	1985	1863 - 1985
Pan22b	Pandora Reef<br>18.815°S, 146.436°E	2005	1974 - 2004
Gfb33a	Geoffrey Bay, Magnetic Island<br>19.15°S, 146.87°E	2012	1987 - 2011

Supplementary References

Bell P, Elmetri I, Uwins P (1999) Nitrogen fixation by Trichodesmium spp. in the Central and Northern Great Barrier Reef Lagoon: relative importance of the fixed-nitrogen load. Marine ecology Progress series 186:119-126

Erler DV, Wang XT, Sigman DM, Scheffers S, Shepherd BO (2015) Controls on the nitrogen isotopic composition of shallow water corals across a tropical reef flat transect Coral Reefs 34:329-338

Furnas M, Alongi D, McKinnon D, Trott L, Skuza M (2011) Regional-scale nitrogen and phosphorus budgets for the northern (14ºS) and central (17ºS) Great Barrier Reef sheld ecosystem. Cont Shelf Res 31:1967-1990

Lough J, Lewis S, Cantin N (2015) Freshwater impacts in the central Great Barrier Reef: 1648–2011. Coral Reefs 34:739-751

Messer LF, Brown MV, Furnas MJ, Carney RL, McKinnon AD, Seymour JR (2017) Diversity and Activity of Diazotrophs in Great Barrier Reef Surface Waters. Frontiers in Microbiology 8

Ren H, Chen Y-C, Wang XT, Wong GT, Cohen AL, DeCarlo TM, Weigand MA, Mii H-S, Sigman DM (2017) 21st-century rise in anthropogenic nitrogen deposition on a remote coral reef. Science 356:749-752

Wang XT, Sigman DM, Cohen AL, Sinclair DJ, Sherrell RM, Cobb KM, Erler DV, Stolarski J, Kitahara MV, Ren H (2016) Influence of open ocean nitrogen supply on the skeletal δ 15 N of modern shallow-water scleractinian corals. Earth Planet Sc Lett 441:125-132

Waters D, Carroll C, Ellis R, Hateley L, McCloskey G, Packett R, Dougall C, Fentie (2013) Modelling reductions of pollutant loads due to improved management practices in the Great Barrier Reef catchments - Whole of GBR, Toowoomba, Queensland
